# BIOMARKERS OF AGING - A CONSORTIUM STORY 

**DOI:** 10.1093/geroni/igaf122.1668

**Published:** 2025-12-31

**Authors:** Mahdi Moqri

**Affiliations:** Harvard University, Cambridge, Massachusetts, United States

## Abstract

The search for biomarkers that quantify biological aging has intensified in recent years. Such biomarkers could predict aging-related outcomes and could serve as surrogate endpoints for the evaluation of interventions promoting healthy aging and longevity. However, no consensus exists on how biomarkers of aging should be validated before their translation to the clinic. I will present the work on the Biomarkers of Aging Consortium over the past three years to address this unmet need.

